# Establishing Magnetic Resonance Imaging as an Accurate and Reliable Tool to Diagnose and Monitor Esophageal Cancer in a Rat Model

**DOI:** 10.1371/journal.pone.0093694

**Published:** 2014-04-04

**Authors:** Juliann E. Kosovec, Ali H. Zaidi, Yoshihiro Komatsu, Pashtoon M. Kasi, Kyle Cothron, Diane V. Thompson, Edward Lynch, Blair A. Jobe

**Affiliations:** 1 Institute for the Treatment of Esophageal and Thoracic Disease, Allegheny Health Network, Pittsburgh, Pennsylvania, United States of America; 2 International Scholars Program, University of Pittsburgh Medical Center, Pittsburgh, Pennsylvania, United States of America; 3 Department of Radiology, Allegheny Health Network, Pittsburgh, Pennsylvania, United States of America; 4 Department of Medicine, Allegheny Health Network, Pittsburgh, Pennsylvania, United States of America; 5 Department of Pathology, Allegheny Health Network, Pittsburgh, Pennsylvania, United States of America; University Hospital Heidelberg, Germany

## Abstract

**Objective:**

To assess the reliability of magnetic resonance imaging (MRI) for detection of esophageal cancer in the Levrat model of end-to-side esophagojejunostomy.

**Background:**

The Levrat model has proven utility in terms of its ability to replicate Barrett’s carcinogenesis by inducing gastroduodenoesophageal reflux (GDER). Due to lack of data on the utility of non-invasive methods for detection of esophageal cancer, treatment efficacy studies have been limited, as adenocarcinoma histology has only been validated post-mortem. It would therefore be of great value if the validity and reliability of MRI could be established in this setting.

**Methods:**

Chronic GDER reflux was induced in 19 male Sprague-Dawley rats using the modified Levrat model. At 40 weeks post-surgery, all animals underwent endoscopy, MRI scanning, and post-mortem histological analysis of the esophagus and anastomosis. With post-mortem histology serving as the gold standard, assessment of presence of esophageal cancer was made by five esophageal specialists and five radiologists on endoscopy and MRI, respectively.

**Results:**

The accuracy of MRI and endoscopic analysis to correctly identify cancer vs. no cancer was 85.3% and 50.5%, respectively. ROC curves demonstrated that MRI rating had an AUC of 0.966 (*p*<0.001) and endoscopy rating had an AUC of 0.534 (*p = *0.804). The sensitivity and specificity of MRI for identifying cancer vs. no-cancer was 89.1% and 80% respectively, as compared to 45.5% and 57.5% for endoscopy. False positive rates of MRI and endoscopy were 20% and 42.5%, respectively.

**Conclusions:**

MRI is a more reliable diagnostic method than endoscopy in the Levrat model. The non-invasiveness of the tool and its potential to volumetrically quantify the size and number of tumors likely makes it even more useful in evaluating novel agents and their efficacy in treatment studies of esophageal cancer.

## Introduction

Treatment options for patients with esophageal adenocarcinoma (EAC) are currently limited and complicated by the fact that most patients present with advanced stage disease [1]–[3]. Furthermore, the prevalence of esophageal adenocarcinoma continues to increase in the United States, and the five-year survival rate is only 15% [4]. It has therefore become increasingly relevant to test new techniques and novel agents to prevent and treat EAC through implementation of comprehensive translational models. The surgical model of end-to-side esophagojejunal anastomosis in rats (modified Levrat model) induces chronic gastroduodenoesophageal reflux (GDER) and exposes the esophagus to gastric acid and bile to initiate disease progression [5]–[10]. The Levrat model has been validated to effectively replicate the same longitudinal progression from gastroesophageal reflux disease (GERD) to Barrett’s esophagus (BE) to EAC that is observed in humans [11], [12]. Additionally, the model is highly efficient, as it has been shown that approximately 70% of rats develop EAC by 28 weeks post-surgery [13].

Previous studies have proven the utility of this model to determine the efficacy of preventative agents against disease progression, but the potential for use as a treatment model has been limited by an inability to reliably diagnose pathology antemortem [13], [14]. Visual endoscopic analysis has been utilized as a tool for monitoring the natural history of disease [15], with biopsy allowing for analysis of molecular markers while avoiding animal sacrifice [16]–[18]; however, due to the inherent model limitation of the extra-luminal presentation of the majority of tumors, as well as the low accuracy of endoscopic biopsies, adenocarcinoma histology has only been validated post-mortem [15].

Alternative imaging techniques such as micro-PET scanning have been explored in an attempt to resolve this issue; however, no technique has been proven significantly sensitive and specific when compared to the gold standard of histology [19]. Recently, magnetic resonance imaging (MRI) has been successfully utilized as a non-invasive imaging method in breast and liver cancer animal models [20]–[22]. With post-mortem histological analysis being the gold standard for EAC, our objective was to examine whether MRI would prove to be a valid tool to detect the presence of esophageal cancer in the Levrat model as compared to visual inspection with endoscopy.

## Materials and Methods

### Levrat Model

**Figure 1:**
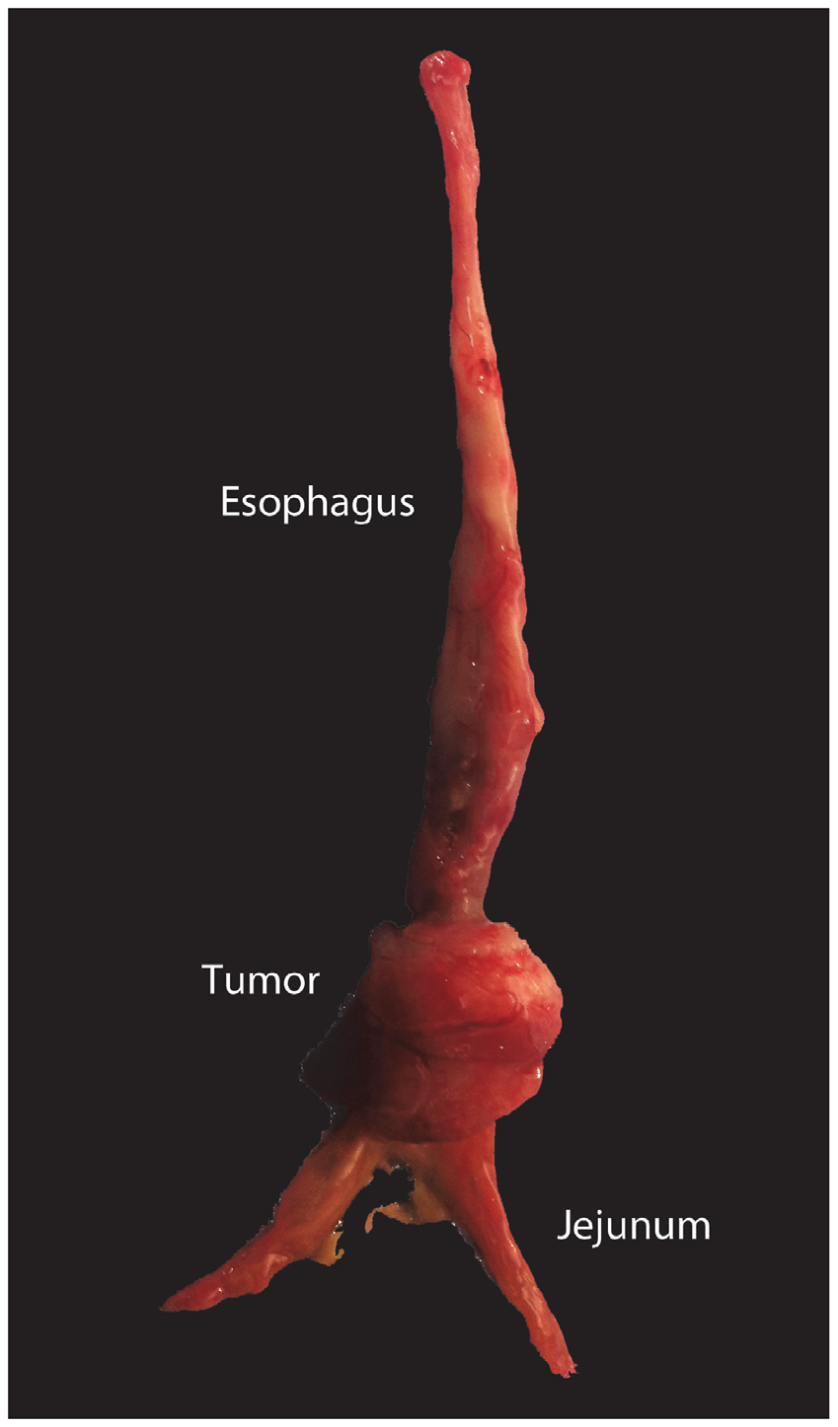
The Levrat Model. Gross image of esophageal tumor (animal no. 13) induced by modified Levrat model.

The Institutional Animal Care and Use Committee of the University of Pittsburgh approved a protocol for the development of this study (protocol no. 1104373). All animals received humane care in compliance with the “Guide for the care and use of laboratory animals.” End-to-side esophagojejunal anastomosis (Fig. 1) was performed on 300 g 6–8 week-old male Sprague-Dawley rats (Harlan Laboratories, Indianapolis, IN) according to the surgical procedure and monitoring standards previously described [13]. The weight and health status of the animals were monitored on a daily basis. Animals were sacrificed prior to the endpoint of the study if they experienced greater than 45% weight loss or acute health concerns and received supplemental diet of mushed pellet or gel diet (Nutra-Gel S5769, BioServ) if weight loss reached greater than 25%. Rats were euthanized at 40-weeks post-operatively through carbon dioxide inhalation for histological evaluation. Animals were scheduled to receive MRI and endoscopy at 32, 36, and 40 weeks post-operatively.

### Endoscopic Assessment

Food and water were restricted from animals for 2–6 hours pre-endoscopic evaluation and for 2–4 hours post-procedure. Rats were anesthetized with isofluorane at 5% and 2% induction and maintenance, respectively. Animals were intubated using an adjusted 16-gauge intravenous catheter to maintain the airway during endoscopy but were not ventilated. Rats were positioned supine on a water-heated surgical bed to maintain body temperature during evaluation and a nose cone was secured using masking tape over intubation tube. Visual endoscopic evaluation was performed of entire esophagus using a mini-rigid, fiber optic light source Hopkins II Forward-Oblique Telescope 30° (diameter 1.9/2.1 mm, length 19 cm), and biopsy specimens were obtained using 1 mm pinch miniature biopsy forceps with double-action jaws (Karl Storz, Tuttlingen, Germany). Air was introduced through side port of endoscope to aid visualization of distal esophagus. Four biopsies were obtained during each evaluation, two for histological evaluation and two for molecular correlate analysis. All endoscopic evaluations were recorded using a digital video recorder (AIDA HD Connect DVD, Karl Storz, Tuttlingen, Germany). Following endoscopic evaluation, animals were injected intramuscularly for days 0 to 3 with ketoprofen (3 mg/kg) and enrofloxacin (5 mg/kg) in an effort to reduce discomfort and prevent respiratory infection from aspiration. Animals were also placed on a three day modified diet progression from gel diet to mushed pellet diet to normal pellet diet, respectively.

### MRI Analysis

All MRI evaluations were performed within 24 hours of endoscopic analysis. Animals were anesthetized with isofluorane at 5% induction and 2% maintainance through nose cone. Rats were placed prone onto the MRI table and secured. A small pressure balloon was placed below thoracic cavity to monitor respiratory rate and 2-lead electrocardiogram electrodes were placed on left front and right back paws to monitor pulse rate (MR-Compatible Model 1030 Monitoring and Gating System, Small Animal Instruments, Inc., Stony-Brook, NY). MRI scans using a T2-weighted respiratory-gated turbo spin-echo (TSE) sequence with an echo time (TE) of 27 msec and repetition time (TR) of approximately 1200–1800 msec were performed using a small animal 7 T ClinScan MRI (Bruker) utilizing Siemens MRI interface (version VB15A). MRI images were acquired in both axial and coronal planes. Acquisition time was 9–10 min per plane.

### Histological Processing and Pathological Evaluation

**Figure 2:**
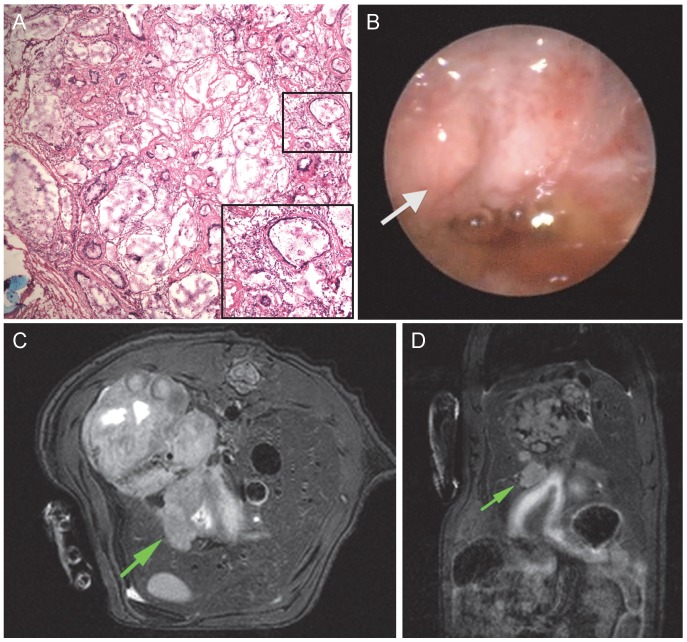
Comparative analysis of a single animal with histology, endoscopy and MRI. A, Hemotoxylin and eosin staining (magnification×10) shows histological confirmation of EAC. B, Endoscopic evaluation of the same rat. Arrow designates suspected area of tumor identified by endoscopic study participants. C, Axial MRI assessment. The arrow shows an abnormal mass on esophageal wall selected by MRI study participants. D, Coronal MRI image. Arrow identifies corresponding cross section of suspected tumor in anastomotic area of interest. MRI; magnetic resonance imaging, EAC; esophageal adenocarcinoma.

Biopsy specimens obtained for histological purposes and to evaluate molecular correlates were snap frozen in OCT compound (Tissue-Tek) and in RLT buffer (Qiagen), respectively. Animals were sacrificed immediately following completion of final endoscopic and MRI evaluations. Upon necropsy, the entire esophagus and jejunum approximately 1 cm distal to anastomosis was collected and opened longitudinally for visual inspection and removal of artifact before specimens were embedded in OCT and snap frozen. Tissue sections were cut (5 μm) from OCT blocks and stained in hematoxylin and eosin (Fig. 2). Two independent experienced pathology experts performed histological analysis. Samples where consensus was not reached, as well as tumors not visible on gross examination (non-protruding; <1 mm diameter) were excluded.

### Design of Blinded Interpretation Study

#### Endoscopy

Endoscopic videos were edited using QuickTime Player (Version 10.1, Apple Inc.) to include complete visualization of distal esophagus (Fig. 2). Videos were cut to exclude biopsy scenes, so as not to bias the observer to potential areas of interest. All videos were imported into a slideshow in triplicate, randomized and de-identified (PowerPoint 2010, Microsoft). Immediately prior to the study, esophageal surgical endoscopists and gastroenterologists were presented with a tutorial outlining the surgical model, aims of the study, and classification of surface mucosal patterns. Endoscopic characteristics were described as the following: *normal* – characterized by smooth surface and even coloration; *esophagitis* – characterized by elevated plaques and ridges and opaque coloration with exudate; *Barrett’s-like-intestinal-metaplasia* – characterized by even-surfaced salmon-colored patches; *cancer* – characterized by irregular elevated masses and/or ulceration. Observers were instructed to grade each video as positive or negative for cancer. Additionally, observers were asked to manually circle suspected areas of positive tumor in the video for the first triplicate subset using PowerPoint Ink Tools. All slideshows were recorded to preserve annotations.

#### MRI

All MRI DICOM images were transferred to OsiriX (version 4.1, Pixmeo Sari) in triplicate, randomized and de-identified for blinded study evaluation. Study participants were presented with a tutorial outlining the surgical model, aims of the study, and classification of pathology as follows: *normal* – even thickness of esophageal wall from proximal to distal esophagus; *benign stricture* – increase in thickness of esophageal wall in distal esophagus with smooth boundaries; – uneven increase in esophageal wall resulting in irregular mass. Radiologist experts were instructed to interpret MRI images correlated in axial and coronal planes and record positive or negative for cancer (Fig. 2). Additionally, participants were asked to circle suspected areas of tumor on the axial images for the first triplicate subset. All images containing annotations were saved.

### Statistical Analysis

Percentage agreement was calculated as the total number of times the rater agreed with histology divided by the total number of readings completed. Cohen’s kappa was used as a measure of agreement between an individual rater and histology diagnoses (e.g., positive or negative cancer). Kappa values were assessed as follows: <0 represent no agreement; between 0.01–0.20 represent slight agreement; between 0.21–0.40, fair agreement; between 0.41–0.60, moderate agreement; between 0.61–0.80 substantial agreement; and between 0.81–0.99 excellent agreement [23]. The intraclass correlation coefficient (ICC) was used to estimate inter and intra-rater reliability for tumor presence/absence made by the esophageal surgical endoscopists and gastroenterologists viewing endoscopy videos and by radiologists reading MRI scans. ICC can range from 0 (no agreement) to 1 (complete agreement). Increasing ICC for both inter and intra-rater reliability indicate increasing agreement. Five individual raters read each rat three times for MRI. Five different raters read each rat three times for endoscopy. A consensus reading (≥2/3) was determined for each rater and each rat. An average of all consensus ratings for all raters was determined for each modality. Sensitivity, specificity, and false negative and false positive values were calculated using histology as the reference standard. A receiver operating characteristic (ROC) curve was constructed for MRI and endoscopy using average consensus rating as the test variable and histology as the state variable. A p-value <0.05 was considered to indicate statistical significance. Data was analyzed using PASW Statistics, version 18.0 (IBM-SPSS, Inc., Chicago).

## Results

### Rats and Histology

A cohort of 38 animals, having undergone the modified Levrat’s surgery, were selected to receive endoscopic and MRI evaluation at 32, 36, and 40 weeks after surgery. Ten animals were removed from the study due to preliminary death (26.3%). Necropsies were formed on all animals and causes of death included: weight loss (n = 5), acute respiratory infection (n = 2), and unknown (n = 3). The reported mortality rate and associated causes of death were consistent with previously reported studies utilizing the Levrat model [13].

Histological evaluation was performed on all remaining 28 study samples and verified by an independent observer. Samples where consensus could not be reached on histology (n = 5) and microscopic tumors (n = 4) were removed from the study cohort, resulting in a final study sample of 19 animals. Of the 19 animals, 57.9% were reported to have EAC (n = 11). There was no significant correlation between endoscopic biopsies and post-mortem histology (data not shown), as most samples were difficult reads due to crush artifact, inadequate depth or indeterminate orientation.

### Endoscopic Analysis

**Figure 3:**
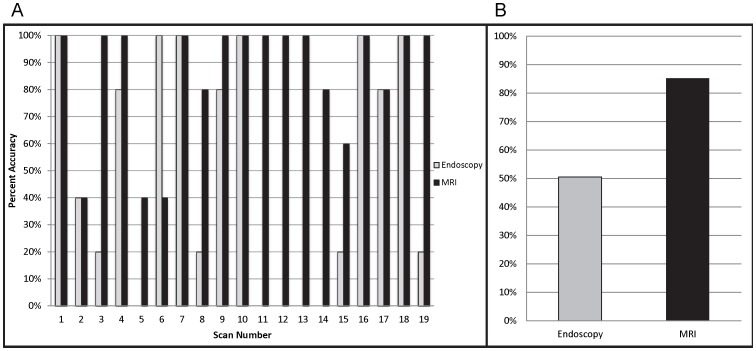
Accuracy assessment of MRI and endoscopy. A, The graph shows percent accuracy of individual scans when assessed by each technique: visual endoscopy and MRI. B, The graph compares average accuracy of each technique as evaluated by the blinded study. MRI; magnetic resonance imaging.

**Figure 4:**
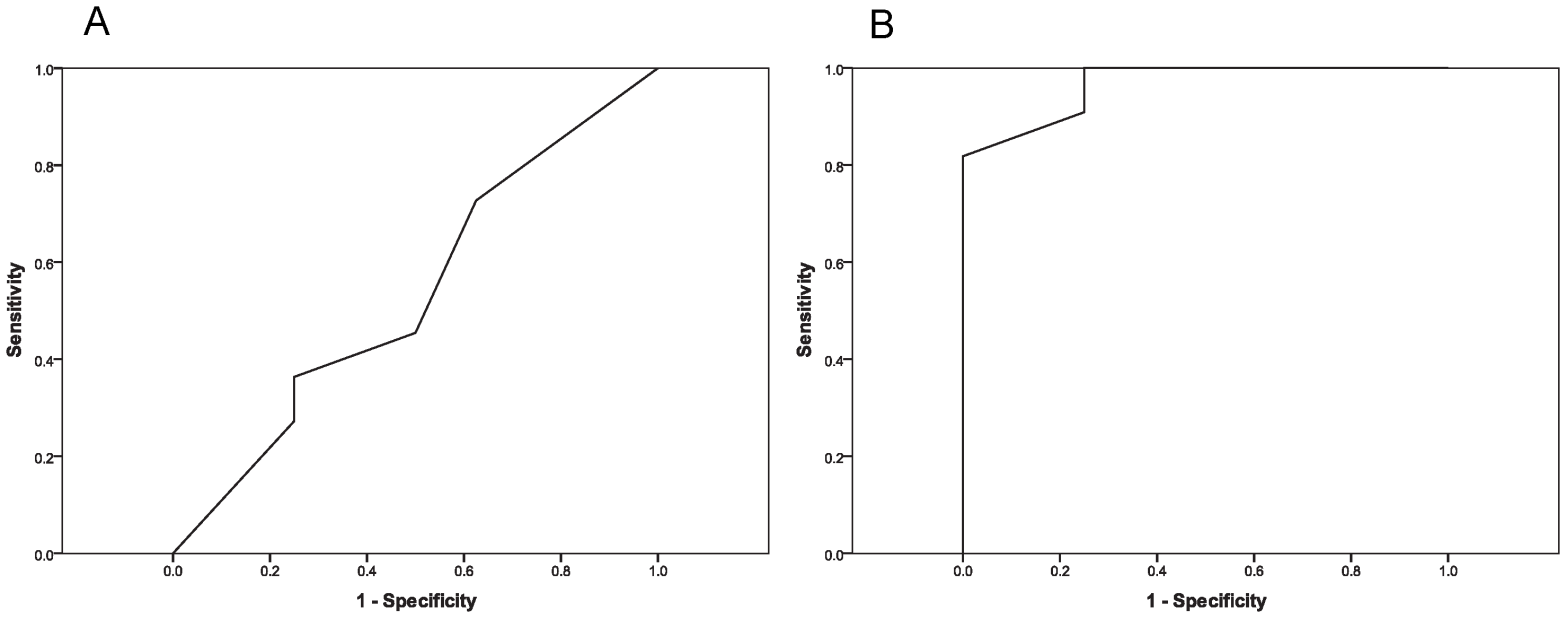
ROC curves of MRI and endoscopic evaluation. A, ROC curve of average endoscopic reading (AUC = 0.534; SE = 0.138; asymptotic significance = 0.80; 95% confidence interval, 0.263–0.805). B, ROC curve of average MRI reading. (AUC = 0.966; SE = 0.036; asymptotic significance <.001; 95% confidence interval, 0–1.0). ROC; receiver operating characteristic, AUC; area under curve, SE; standard error.

Of the 5 blinded study participants, percentage agreement with histology ranged between 42.1%–57.9%, with an average agreement of 50.5% (Fig. 3). Kappa values for individual raters fell between −0.044–0.165, and no evaluations reached statistical significance (p-value >0.05) (Table 1). 47.37% of scans could be read with at least 80% accuracy (9/19) (Fig. 3). Intra-rater reliability ICCs ranged between 0.667–0.873 among triplicate scans; whereas inter-rater ICC was 0.608. ROC curve analysis produced an area under the curve (AUC) of 0.534 (SE = 0.138; asymptotic significance = 0.804; 95% confidence interval, 0.263–0.805) (Fig. 4). Sensitivity of endoscopy was 0.455 (95% confidence interval, 0.323–0.586), specificity was 0.575 (95% confidence interval, 0.422–0.728) and diagnostic accuracy (efficiency) was 0.505 (95% confidence interval, 0.405–0.606). Average false positive and average false negative rates were 0.425 (95% confidence interval, 0.272–0.578) and 0.546 (95% confidence interval, 0.0.414–0.679), respectively. Even when 5/5 endoscopy evaluators rated a tumor as positive, there was a 40% false positive rate.

**Table 1:** Statistical evaluation of individual participants in blinded study evaluating MRI and endoscopy.

		Kappa			
Rater	Agreement with Histology	K	95% CI	Significance	Intra-Rater Variability(ICC with 95% CI)
**MRI**					
1	89.47% (17/19)	0.791	0.524–1.00	**<.001**	0.578 (0.315–0.790)
2	78.95% (15/19)	0.553	0.176–0.930	**0.013**	0.594 (0.335–0.799)
3	84.21% (16/19)	0.671	0.331–1.00	**.003**	0.708 (0.487–0.863)
4	94.74% (18/19)	0.890	0.682–1.0	**<.001**	0.312 (0.032–0.609)
5	78.95% (15/19)	0.568	0.193–0.941	**0.013**	0.785 (0.603–0.902)
**Endoscopy**					
1	52.63% (10/19)	.045	−0.402–0.492	**0.845**	0.873 (0.752–0.944)
2	47.37% (9/19)	−0.044*	−0.484–0.396	**0.845**	0.667 (0.429–0.841)
3	57.89% (11/19)	0.165	−0.268–0.598	**0.463**	0.791 (0.613–0.905)
4	42.11% (8/19)	−0.01*	−0.422–0.401	**0.960**	0.781 (0.597–0.901)
5	52.63% (10/19)	−0.01*	−0.422–0.401	**0.960**	0.676 (0.442–0.846)

*Negative values indicate less than chance agreement.

### MRI Evaluation

Percentage agreement with histology of MRI participants ranged between 78.95%–94.7% with an average agreement of 85.3% (Fig. 3) and kappa values of individual rates between 0.553–0.890. All raters reached statistical significance (p-value >0.05) (Table 1). 78.95% of scans could be read with at least 80% accuracy (15/19) (Fig. 3). Intra-rater reliability ICCs among triplicate scans fell between 0.315–0.785 and inter-rater ICC was 0.624. ROC curve analysis determined an AUC of 0.966 (SE = 0.035; asymptotic significance <0.001; 95% confidence interval, 0–1.00) with sensitivity of 0.891 (95% confidence interval, 0.809–0.973), specificity of 0.800 (95% confidence interval, 0.676–0.924), and efficiency of 0.853 (95% confidence interval, 0.781–0.924) (Fig. 4). Average false positive and false negative rates were 0.200 (95% confidence interval, 0.076–0.324) and 0.109 (95% confidence interval, 0.027–0.208), respectively. With an average MRI rating of 0.80 or greater (4/5 or 5/5 ratings positive for tumor), MRI average rating was noted to be predictive of tumor with no false positives.

## Discussion

The search for new treatment methods for EAC has become extremely important in recent years with the rise of patients presenting with esophageal cancer. [24], [25]. Since MRI had recently been utilized successfully in small animal translational models for other types of cancers [20]–[22], we aimed to test the ability of MRI to effectively diagnose esophageal tumor in the Levrat model as compared to endoscopy. As noted, we found MRI to be a very valid and reliable method for detection of esophageal tumors as compared to endoscopy. Though endoscopic evaluation has its own advantages, in our study, biopsies could not provide reliable histology due to the small sample size and complicated orientation.

When compared to endoscopic evaluation, MRI was noted to be superior in detection of adenocarcinoma on all levels, including accuracy, sensitivity, specificity, and efficiency. Inter-observer consensus was also higher for MRI indicating future evaluations would likely produce similar results. Intra-observer consensus among triplicate readings was the only variable where endoscopic values represented a higher range, indicating increased consensus among endoscopic readings. Still, MRI scans with the lowest levels of intra-observer consensus were consistent with scans producing lowest accuracy levels, indicating variability was mostly limited to difficult scans. There were four cases where the average accuracy of MRI scans was noted to be below 80%. In three of the cases, the interpretation was difficult due to the presence of an abscess at the anastomotic site or the stomach; whereas in the fourth case, there was significant motion effect.

The non-invasive nature of the MRI, its ability to accurately identify cancer, and incorporation into the Levrat model makes the modality even more applicable to evaluate novel agents and their efficacy in treatment studies. The high accuracy and low false positive rates indicate MRI would be a reliable method to differentiate cancer vs. no cancer treatment arms with minimal error. Additionally, the low false negative rate would also help in maximizing efficiency and minimizing the amount of animals needed for a particular protocol. On the other hand, endoscopy produced a 40% false positive rate, even when all endoscopic experts agreed tumor was present. Therefore, visual endoscopy would be a poor discriminator of tumor status for such a study.

Limitations of our study include the small sample size. In addition, although the specific sequence utilized in this study was able to accurately detect the presence of the tumor, the entire volume of suspicious masses was not quantified due to the inherent 2-dimensional nature of the TSE scan. Future studies may benefit from a 3-dimensional scan. A 3-D MRI sequence integrates the axial, coronal, and sagittal planes to allow for accurate volumetric quantification of anatomical features. In the outlined model, a 3-D MRI scan could be utilized to analyze efficacy in a treatment model by providing tumor volume pre- and post-treatment. Utilization of contrast agents to enhance the anastomotic area of interest and differentiate tumor would further increase the accuracy of this modality.
